# ADAM17-dependent proteolysis of L-selectin promotes early clonal expansion of cytotoxic T cells

**DOI:** 10.1038/s41598-019-41811-z

**Published:** 2019-04-02

**Authors:** Rebar N. Mohammed, Sophie C. Wehenkel, Elena V. Galkina, Emma-Kate Yates, Graham Preece, Andrew Newman, H. Angharad Watson, Julia Ohme, John S. Bridgeman, Ruban R. P. Durairaj, Owen R. Moon, Kristin Ladell, Kelly L. Miners, Garry Dolton, Linda Troeberg, Masahide Kashiwagi, Gillian Murphy, Hideaki Nagase, David A. Price, R. James Matthews, Vera Knäuper, Ann Ager

**Affiliations:** 1 Divsion of Infection and Immunity, School of Medicine, Cardiff University, Cardiff, CF14 4XN UK; 2 College of Veterinary Medicine, University of Sulaimani, Sulaimani, Kurdistan Iraq; 3 Francis Crick Institute, London, NW1 1AT UK; 4 Department of Microbiology and Molecular Cell Biology, Eastern Virginia Medical School, Norfolk, VA 23507 USA; 5 Norwich Medical School, University of East Anglia, Norwich, NR4 7UQ UK; 6 Takeda Pharmaceutical Research Institute, Tsukuba, Japan; 7 University of Cambridge Depratment of Oncology, Cancer Research UK Cambridge Insitute, Li Ka Shing Centre, Cambridge, CB2 0RE UK; 8 Kennedy Institute of Rheumatology, University of Oxford, Oxford, OX3 7FY UK; 9 Systems Immunity Research Institute, Cardiff University, Heath Park, Cardiff CF14 4XN UK; 10 School of Dentistry, Cardiff University, Heath Park, Cardiff CF14 4XN UK

## Abstract

L-selectin on T-cells is best known as an adhesion molecule that supports recruitment of blood-borne naïve and central memory cells into lymph nodes. Proteolytic shedding of the ectodomain is thought to redirect activated T-cells from lymph nodes to sites of infection. However, we have shown that activated T-cells re-express L-selectin before lymph node egress and use L-selectin to locate to virus-infected tissues. Therefore, we considered other roles for L-selectin proteolysis during T cell activation. In this study, we used T cells expressing cleavable or non-cleavable L-selectin and determined the impact of L-selectin proteolysis on T cell activation in virus-infected mice. We confirm an essential and non-redundant role for ADAM17 in TCR-induced proteolysis of L-selectin in mouse and human T cells and show that L-selectin cleavage does not regulate T cell activation measured by CD69 or TCR internalisation. Following virus infection of mice, L-selectin proteolysis promoted early clonal expansion of cytotoxic T cells resulting in an 8-fold increase over T cells unable to cleave L-selectin. T cells unable to cleave L-selectin showed delayed proliferation *in vitro* which correlated with lower CD25 expression. Based on these results, we propose that ADAM17-dependent proteolysis of L-selectin should be considered a regulator of T-cell activation at sites of immune activity.

## Introduction

L-selectin delivers naïve and central memory T-cells from the bloodstream into lymph nodes to survey antigen presenting cells (APC) for peptide-MHC complexes. It has long been known that L-selectin is proteolytically shed from the T-cell surface within hours following engagement of the T-cell receptor (TCR)^1^ and that lack of L-selectin expression is a characteristic feature of effector and effector memory T cells inside inflamed and infected tissues^2^. These findings have suggested that downregulation of cell surface L-selectin is required to prevent activated T-cells re-entering lymph nodes from the bloodstream and allow entry into infected and inflamed tissues. However, we have shown that, following downregulation of L-selectin by peptide-MHC complexes inside lymph nodes, L-selectin is fully re-expressed on virus-specific early effector CD8^+^ T cells before they egress lymph nodes^3^. Moreover, re-expressed L-selectin is essential for circulating effector T cells to home to and clear virus from infected organs. If L-selectin downregulation is not required to re-direct activated T-cells to sites of inflammation, what is the role of L-selectin proteolysis during T cell activation?

Cross-linking of L-selectin primes T-cells for antigen-induced proliferation^4^ and controls important effector functions such as superoxide production^5^, colony-stimulating factor 1 release^6^ and lytic activity^7^. The cytoplasmic tail of L-selectin is phosphorylated by non-receptor kinases bound via adapter proteins following ligand engagement and phosphorylation is linked to effector activities^5,6^. It is reasonable to propose that TCR-induced proteolytic shedding of the ectodomain of L-selectin will abrogate signalling initiated and sustained by ligand binding. However, TCR engagement also stimulates phosphorylation-dependent binding of protein kinase C isozymes ι, θ, and α to the cytoplasmic tail of L-selectin^8^. It is, therefore, possible that the transmembrane fragment of L-selectin with bound signalling complexes left after TCR-induced shedding of the ectodomain has the potential to move into different cellular compartments to propagate, rather than abrogate, L-selectin-dependent signalling.

The metalloproteinase disintegrins ADAM10 and ADAM17 have emerged as important enzymes controlling ectodomain shedding of multiple substrates in haemopoietic and non-haemopoietic cells, particularly in response to cellular activation by ionomycin and phorbol esters respectively^9^. Studies of mice with selective inactivation of *adam17* in leucocytes, T cells or B cells have shown a dominant role for ADAM17 in shedding of L-selectin stimulated by phorbol esters^9–13^. Moreover, ADAM17 deficient T cells are unable to shed L-selectin early after activation by anti-CD3 antibodies^13^. However, ADAM17 deficient T cells are not ideal for studying the role of L-selectin proteolysis in T cell activation for several reasons. Firstly, enzymes other than ADAM17 cleave L-selectin since plasma levels of shed L-selectin are not altered in mice selectively deficient in leucocyte ADAM17^11^. Secondly, substrates of ADAM17 other than L-selectin that are proteolytically shed following TCR activation have already been shown to control T cell proliferation and/or differentiation, such as IL6Rα^13^ and LAG-3^14^. Thus, although L-selectin may not be proteolyzed, the lack of proteolysis of other important regulators of T cell activation may mask any role for L-selectin proteolysis in ADAM17 null T cells.

To study the role of L-selectin proteolysis directly, we exploited T-cells expressing a metalloprotease cleavage-resistant mutant of L-selectin to determine the impact of TCR-induced proteolysis of L-selectin on T cell activation during virus infection. Our data show that TCR-induced proteolysis of L-selectin by ADAM17 did not affect early activation of T cells measured by CD69 expression but promoted early clonal expansion of cytotoxic T-cells which correlated with upregulation of CD25.

## Results and Discussion

### ADAM17 is essential for TCR-induced ectodomain proteolysis of L-selectin

**Figure 1:**
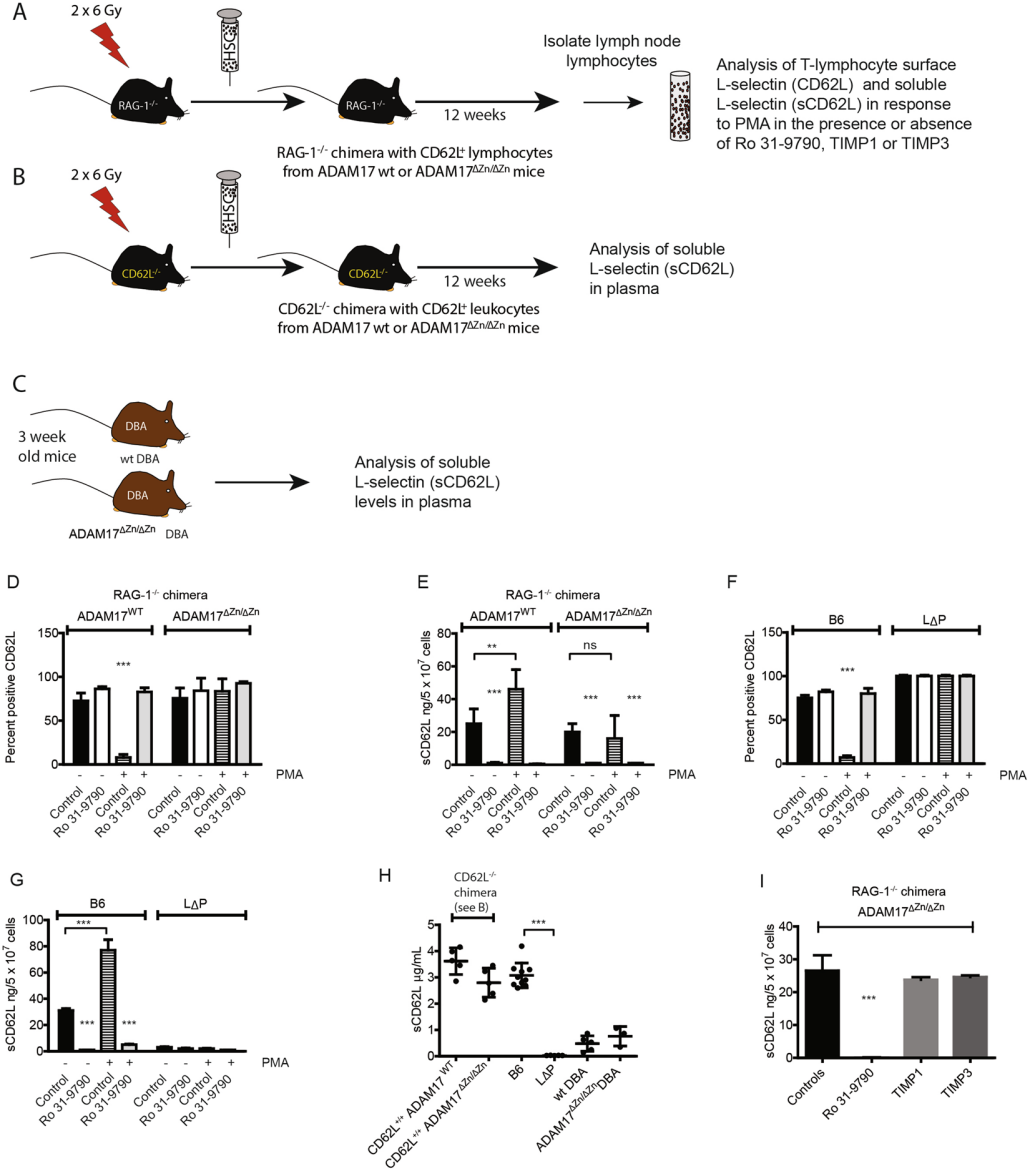
ADAM17-independent shedding of L-selectin in T-cells. (**A–C**) ADAM17 deficient mouse strains used; (**A**) RAG-1^−/−^ radiation chimeras for L-selectin expression in isolated T lymphocytes, (**B**) L-selectin^−/−^ radiation chimeras and (**C**) DBA mice for soluble L-selectin in blood. (**D–G**) Constitutive and activation induced shedding of L-selectin. (**D**,**E**) Lymphocytes from ADAM17-sufficient (ADAM17^WT^) or ADAM17-deficient (ADAM17^ΔZn/ΔZn^) RAG-1^−/−^ chimeras were incubated in the presence of 30 µM Ro 31–9790 and/or 300 nM PMA or vehicle controls (Control) and analyzed for (**D**) T cell expression of L-selectin (CD62L) (see Fig. 1S for gating of CD62L positive T-cells) and (**E**) shed L-selectin. (**F**,**G**) Lymphocytes from B6 and L∆P mice were incubated in the presence of 30 µM Ro 31–9790 and/or 300 nM PMA or vehicle controls (Control) and analyzed for (**F**) T cell expression of L-selectin (CD62L) and (**G**) shed L-selectin (sCD62L). (**H**) Soluble L-selectin in plasma from ADAM17^WT^ or ADAM17^ΔZn/ΔZn^ L-selectin^−/−^ chimeras, adult B6, adult LΔP, 3-week old DBA mice and 3-week old ADAM17^ΔZn/ΔZn^ DBA mice was analyzed by ELISA. (**I**) Soluble L-selectin released by ADAM17-deficient lymphocytes in the absence or presence of 30 µM Ro 31–9790, 1 µM TIMP1, or 1 µM TIMP3 was analyzed by ELISA. Bar charts show mean ± SEM (n = 4–10 in **A–D**, n = 4 in **F**). Symbols in panel H show data from individual mice, and horizontal bars indicate mean values. Statistical analysis used two-way ANOVA with Tukey’s post-hoc test in panels (D–G) student’s t test in panel (H) and one-way ANOVA in panel (I). ***P < 0.001.

We aimed to study the role of L-selectin proteolysis in controlling T cell activation during virus infection. Therefore, we started by determining the role of ADAM17 in ectodomain shedding of L-selectin in T cells following activation by virus derived peptide-MHC complexes on antigen presenting cells. Embryos die *in utero* in C57BL/6 (B6) mice lacking ADAM17^10^. However, radiation chimeras reconstituted with ADAM17 deficient haempoietic stem cells are viable^11^. To generate mice in which *adam17* is selectively inactivated in lymphocytes, lethally irradiated, recombination activation gene-1 deficient (RAG-1^−/−^) mice were injected with day 17 foetal liver cells from either ADAM17 deficient (ADAM17^∆Zn/∆Zn^) or ADAM17 sufficient (ADAM17^WT^) embryos (Fig. 1A). Donor-derived lymphocytes were analysed 12 weeks later for spontaneous (constitutive) and phorbol-ester induced shedding of L-selectin to confirm ADAM17 status. Lymphocyte ADAM17 was not essential for reconstitution of lymphocytic lineages as found previously using ADAM17 deficient haemopoietic stem cell chimeric mice^11^. Lymph node cellularities in ADAM17 sufficient (ADAM17^WT^) and ADAM17 deficient (ADAM17^∆Zn/∆Zn^) chimeras were 26 ± 6 × 10^6^ and 23 ± 7 × 10^6^ respectively and T:B lymphocyte ratios, 1.8 ± 0.3 and 2.1 ± 0.2 respectively (mean ± SD, n = 6). ADAM17 did not affect the frequency of L-selectin (CD62L)^+^ T-cells in lymph nodes (Fig. 1D) or L-selectin levels per cell (ADAM17^∆Zn/∆Zn^: ADAM17^WT^, MFI of 1.20 ± 0.29; n = 6). Low levels of constitutive shedding from isolated lymph node lymphocytes^15^ is not dependent on ADAM17 since the levels of soluble (sCD62L) and cell surface L-selectin (CD62L) were not affected by lack of ADAM17 expression (Fig. 1D,E). However, the hydroxamic acid based metalloproteinase inhibitor Ro 31–9790 completely blocked release of soluble L-selectin from both ADAM17^WT^ and ADAM17^∆Zn/∆Zn^ lymphocytes (Fig. 1E) and increased the fraction of L-selectin^+^ T-cells by 15.5 ± 4.0% for ADAM17^WT^ and 19.0 ± 2.0% for ADAM17^∆Zn/∆Zn^ (n = 6). The role of ADAM17 in controlling cell surface expression of L-selectin appears to depend on the T cell source as well as the mouse model; radiation chimeras reconstituted with ADAM17 deficient haemopoietic stem cells showed 3-fold higher cell surface levels of L-selectin on peripheral blood T cells^11^, whereas inactivation of *adam17* did not increase L-selectin levels on T cells isolated from lymphoid tissues^13^, as we have found. However, ADAM17 was essential for phorbol ester induced shedding of L-selectin in T cells as reported previously^10,13^. Phorbol 12-myristate 13-acetate (PMA) had no effect on cell surface or soluble L-selectin levels in ADAM17-deficient T-cells, whereas cell surface L-selectin was reduced from 72.7 ± 3.1 to 7.9 ± 1.7% (n = 4) (Fig. 1D) and soluble L-selectin increased 2-fold in ADAM17-sufficient T-cells (Fig. 1E). Substituting the juxtamembrane L-selectin cleavage site with the homologous region of P-selectin (L∆P) completely inhibited constitutive and PMA-induced L-selectin shedding in isolated lymphocytes (Fig. 1F,G) as reported previously^15^, indicating that ADAM17-dependent and ADAM17-independent L-selectin cleavage occurs within this region.

L-selectin is shed from lymphocytes as well as other types of leucocyte^15–17^ and is detectable in the blood of naïve mice. To determine if ADAM17 controls shedding of L-selectin in mice, we measured soluble L-selectin in peripheral blood. To avoid detection of soluble L-selectin from host-derived leucocytes, we used L-selectin^−/−^ mice as hosts for ADAM17 deficient or ADAM17 sufficient stem cells (Fig. 1B; see Methods). In L-selectin^−/−^ chimeras, the source of soluble, shed L-selectin in blood is restricted to the progeny of injected stem cells. Twelve weeks after reconstitution, circulating levels of soluble L-selectin were not statistically significantly different in ADAM17 sufficient and ADAM17 deficient L-selectin knockout (CD62L^−/−^) chimeras (Fig. 1H). Similar findings have been reported in ADAM17 sufficient and ADAM17 deficient B6 radiation chimeras^11^. These results demonstrate clearly that soluble L-selectin is not generated by ADAM17 expressed by leucocytes, however, it is dependent on metalloproteinase-dependent cleavage as shown by its’ absence in L∆P mice (Fig. 1H). The possibility that ADAM17 on stromal cells generates soluble L-selectin was addressed using a different mouse strain (Fig. 1C). ADAM17 deficiency does not cause embryonic lethality in the DBA strain and mice survive to ~3 weeks of age. This enabled us to determine if L-selectin is shed in the complete absence of ADAM17 from leucocytes as well as stromal cells. Although L-selectin levels were lower in 3-week old mice, the levels were not significantly different in ADAM17 sufficient and ADAM17 deficient DBA mice (Fig. 1H). These findings show clearly that, in the absence of ADAM17 on leucocytes, ADAM17 on stromal cells does not generate soluble L-selectin. Moreover, these data show that metalloproteinases other than ADAM17 can generate soluble L-selectin in mice. *In vitro* studies have shown that ADAM10 cleaves L-selectin in cell lines lacking ADAM17^9^ and that ADAM10 is constitutively active in T cells^14,18^. To determine if ADAM10 substitutes for ADAM17 in T cells, we tested tissue inhibitor of metalloproteinase (TIMP)-1^19^ and TIMP-3^20^, both of which block ADAM10^21^. Neither TIMP-1 nor TIMP-3 blocked constitutive metalloproteinase dependent shedding of L-selectin from ADAM17-deficient T-cells (Fig. 1I), although TIMP-3 inhibited PMA-induced shedding from ADAM17 sufficient mouse T cells^22^. The relationship between plasma L-selectin and L-selectin spontaneously shed from isolated T cells is not completely understood^11,23^. We have shown that soluble and cell surface L-selectin are linked in transgenic mice expressing different levels of L-selectin at the T cell surface^24^, but the stimulus for shedding of L-selectin from T cells in naïve mice is not known. Together, our studies show that blood levels of L-selectin in mice are completely independent of ADAM17 expressed either by leucocytes or by stromal cells. Furthermore, constitutive shedding in isolated T-cells is independent of ADAM17 and ADAM10. Further studies will be required to identify the sheddase or sheddases responsible and determine how their activities are regulated.

**Figure 2:**
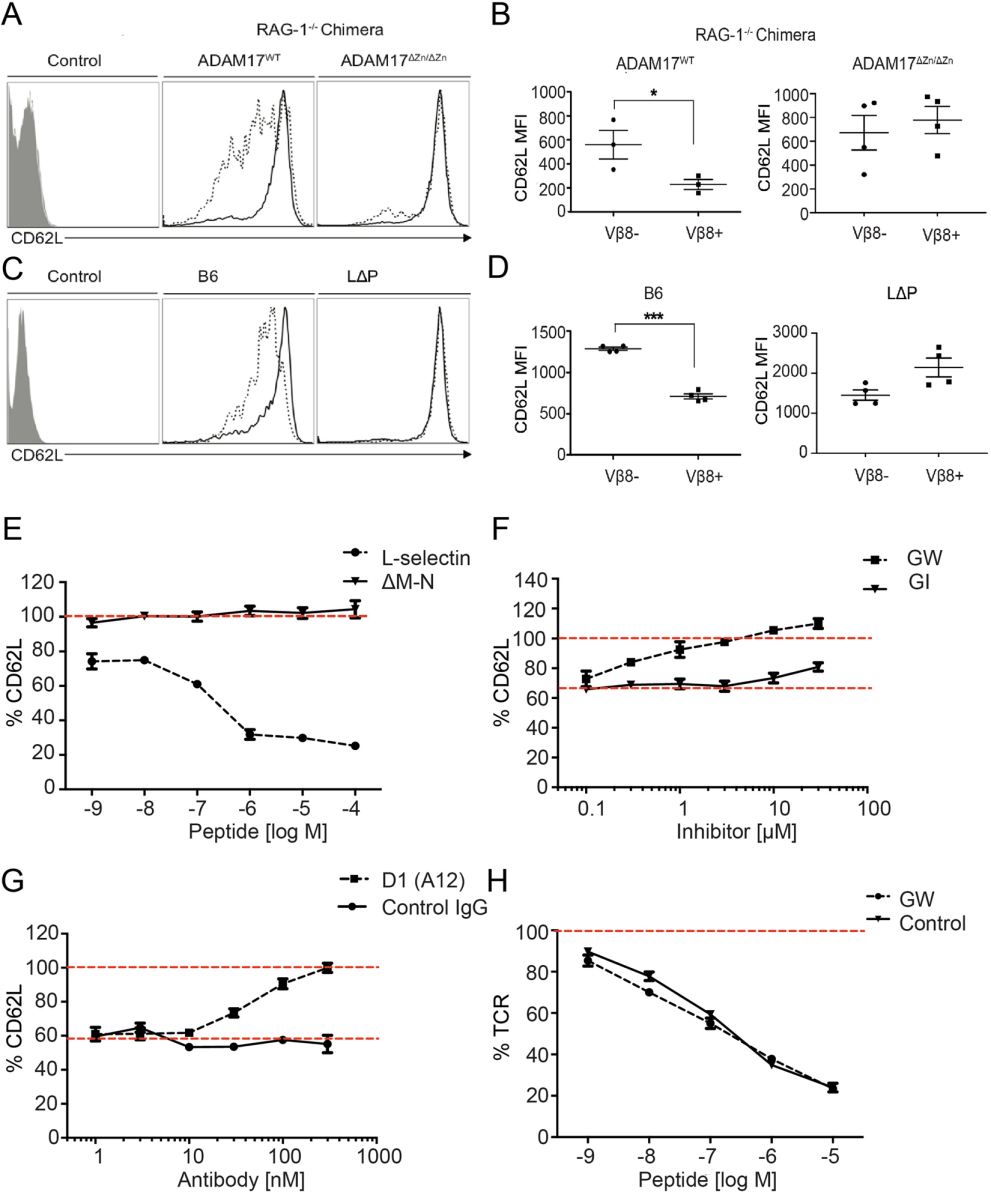
TCR-induced L-selectin downregulation on T-cells requires ADAM17. (**A–D**) ADAM17^WT^, ADAM17^ΔZn/ΔZn^ RAG-1^−/−^ chimeras (**A,B**), B6 and L∆P mice (**C,D**) were injected intraperitoneally with 10 µg of SEB. After 4 h, L-selectin expression on Vβ8^+^ and Vβ8^−^ T cells isolated from lymph nodes was determined by flow cytometry. Representative histograms show L-selectin expression on Vβ8^+^ (dashed line) and Vβ8^−^ (solid line) T-cells versus an isotype control (control) (**A**,**C**). Scatter plots show mean ± SEM (n = 3–5 mice) (**B**,**D**). (**E–G**) Cell surface levels of L-selectin on flow-sorted 868 TCR^+^ Molt3 cells expressing wildtype (**E**–**G**) or ΔM-N (**E**) L-selectin were determined by flow cytometry after incubation for 1 h with SLY peptide-pulsed antigen-presenting cells at a ratio of 1:3. SLY peptide stimulation was conducted in the absence of inhibitors (**E**), the presence of selective ADAM10 inhibitor GI or dual ADAM10/ADAM17 inhibitor GW (**F**), or the presence of blocking ADAM17 antibody D1(A12) or control human IgG (**G**). (**H**) TCR downregulation on 868 TCR^+^ Molt3 cells was determined by flow cytometry in the absence or presence of 30 μM GW. Percentages for L-selectin and TCR expression were obtained by subtracting the median fluorescence intensity (MFI) of the isotype-matched control from the MFI of each sample and normalizing to non-incubated cells stored on ice (100% expression). Cells were gated as live, single lymphocytes, and antigen-presenting cells were excluded using CD19 expression. Red dashed lines indicate 100% expression (**E**–**H**) and maximal downregulation (**F**,**G**). Symbols in panels (B) and (D) show data from individual mice, and horizontal bars indicate means. Results in panels (E–H) are mean ± SEM (n = 3–5). Statistical analysis used unpaired Student’s 2-tailed t test. *P < 0.05; ***P < 0.001.

To determine the role of ADAM17 in TCR induced L-selectin shedding in mice, we used the bacterial superantigen staphylococcus enterotoxin B (SEB), which selectively activates T-cells expressing the Vβ8^+^ chain of the TCR^25^. L-selectin was downregulated on Vβ8^+^ but not Vβ8^−^ T-cells in B6 mice as well as ADAM17-sufficient RAG-1^−/−^ chimeras (Fig. 2A–D). In contrast, SEB did not downregulate L-selectin on ADAM17-deficient Vβ8^+^ T-cells in RAG-1^−/−^ chimeras (Fig. 2A,B). Vβ8^+^ T-cells in transgenic L- selectin (LΔP) mice did not shed L-selectin in response to SEB (Fig. 2C,D); the slight trend to increased expression over Vβ8^−^ T-cells may reflect activity of the heterologous promoter which drives transgene expression. These results demonstrate clearly a non-redundant role for T cell expressed ADAM17 in L-selectin ectodomain proteolysis following TCR activation.

TCR-induced shedding of L-selectin in human T cells was studied using L-selectin deficient Molt-3 T-leukemic cells, transduced to express an HLA-A2-restricted HIV-1 Gag SLYNTVATL (SLY)-specific TCR (868) and either wildtype human L-selectin (L-selectin) or cleavage resistant human L-selectin (ΔM-N)^26^. Constitutive shedding of wildtype L-selectin, but not ΔM-N-L-selectin, was evident by lower expression of L-selectin in unstimulated cells (Fig. 2E). Engagement of the 868 TCR with SLY peptide-MHC stimulated dose-dependent downregulation of L-selectin by 30–40%, but not ΔM-N-L-selectin (Fig. 2E). The roles of ADAM17 and ADAM10 in TCR-induced L-selectin shedding in human cells were dissected by comparing the hydroxamate-based metalloproteinase inhibitor GW280264X (GW) which inhibits both ADAM17 and ADAM10 and GI254023X (GI) which is 100-fold more selective for ADAM10 than ADAM17^27^. These compounds have previously been used to dissect the roles of ADAM10 and ADAM17 in PMA-induced shedding of L-selectin in mouse T cells^12^. GW completely blocked TCR-induced L-selectin shedding at 3 µM, whereas 3 µM GI had no effect (Fig. 2F). The lack of effect of GI at equivalent doses to effective levels of GW indicates that ADAM10 does not substitute for ADAM17 in TCR induced shedding of L-selectin. However, GW and GI inhibit a number of other metalloproteinases^27^, some of which have been shown to shed L-selectin^28^. Therefore, GW cannot be used in isolation to measure ADAM17 activity. An essential role for ADAM17 in TCR-induced shedding of L-selectin in human T cells was demonstrated using the inhibitory anti-ADAM17 antibody D1(A12)^29^ which completely inhibited L-selectin shedding at ≥300 nM (Fig. 2G). Collectively, these results demonstrate an essential role for ADAM17 in TCR-induced L-selectin shedding in T-cells. A role that cannot be substituted for either by ADAM10 or by the constitutively active metalloproteinase in T cells reported herein. There was no evidence of cross-talk between ADAM17-dependent L-selectin proteolysis and early T-cell activation, since TCR downregulation was independent of metalloproteinase inhibition using GW or ΔM-N-L-selectin expression (Fig. 2H; data not shown).

### Ectodomain proteolysis of L-selectin controls rapid clonal expansion of cytotoxic T-cells

**Figure 3:**
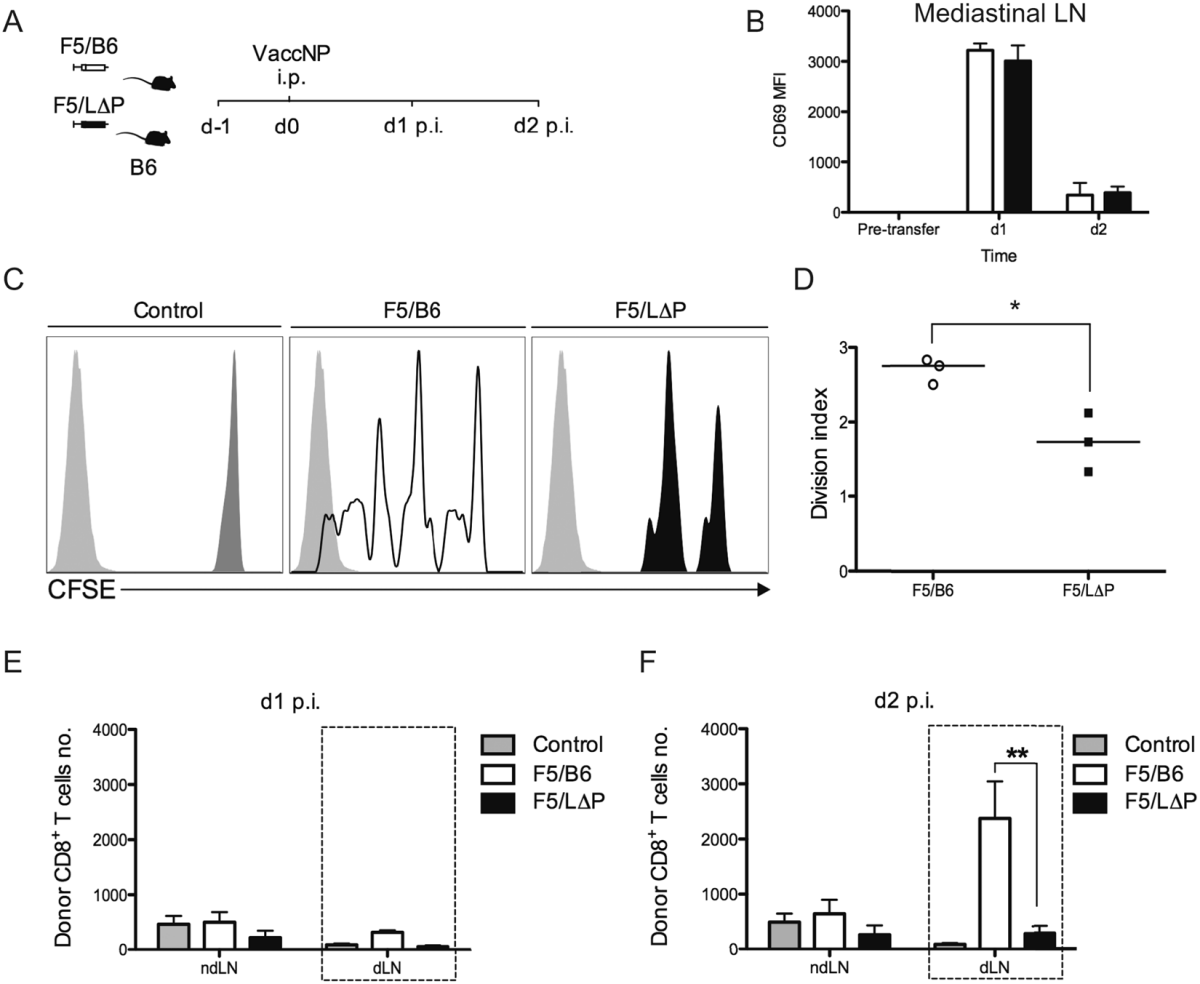
TCR-induced L-selectin shedding promotes the clonal expansion of effector T cells in virus-infected mice. (**A**) Thy1.2 CD8^+^ T cells co-expressing the F5 transgenic TCR and either wildtype (F5/B6) or shedding-resistant (F5/L∆P) L-selectin were labelled with CFSE and injected intravenously into naïve Thy1.1 mice. After 24 hours, mice were inoculated intraperitoneally with vaccNP. Non-draining inguinal lymph nodes (ndLN) and draining mediastinal lymph nodes (dLN) were harvested at days 1 and 2 after virus challenge, and donor cells were analyzed for CD69 expression and dilution of CFSE. (**B**) Bar charts show CD69^+^ F5/B6 and F5/L∆P CD8^+^ T cells in the draining mediastinal lymph nodes at days 1 and 2 post-infection (mean ± SEM, n = 3). (**C**) Representative histograms show CFSE label in F5/B6 and F5/LΔP CD8^+^ T cells in the draining mediastinal lymph nodes of uninfected mice (control) and infected mice at day 2 after inoculation with vaccNP. Unlabelled cells are shown as light grey histograms. (**D**) Scatter plots of division indices for F5/B6 and F5/LΔP CD8^+^ T cells at day 2 after infection with vaccNP. Symbols represent individual mice, and lines indicate mean values. (**E,F**) Bar charts show total numbers of donor F5/B6 and F5/LΔP CD8^+^ T cells in draining mediastinal lymph nodes and non-draining inguinal lymph nodes at days 1 (**E**) and 2 (**F**) after infection with vaccNP. Uninfected mice injected with F5 transgenic CD8^+^ T cells are shown for comparison (control). Results are shown as mean ± SEM (n = 3). Statistical significance was assessed using unpaired Student’s 2-tailed t test in panels (D) and two-way ANOVA with Tukey’s post-hoc test in panel (E). *P < 0.05; **P < 0.01.

CD8^+^ T-cells differentiate into cytotoxic T-cells (CTLs) and initiate rapid clonal expansion in lymphoid organs of mice during the first 24–48 h following virus infection^30^. We have shown that TCR-induced shedding of L-selectin occurs in lymph nodes draining the site of virus administration within 24–48 hours^3^. Therefore, we started by using mouse models of virus infection to determine whether L-selectin proteolysis regulates clonal expansion of cytotoxic T-cells *in vivo*. Naïve CD8^+^ T cells expressing H2D^b^ restricted influenza A nucleoprotein peptide 366–374 (NP68)-specific TCR (F5) and either wildtype L-selectin (F5/B6) or shedding-resistant L-selectin (F5/L∆P) were CFSE labelled and adoptively transferred into naïve B6 mice. After 24 h, recombinant vaccinia virus expressing NP68 (vaccNP) was injected intraperitoneally, and the draining mediastinal lymph nodes harvested on days 1 and 2 post-infection for analysis of donor CD8^+^ T-cell activation and proliferation (Fig. 3A). L-selectin proteolysis did not affect CD8^+^ T-cell priming in response to stimulation with peptide-MHC complexes on antigen-presenting cells, as CD69 was upregulated at day 1 post-infection and downregulated at day 2 post-infection to a similar extent on F5/B6 and F5/LΔP CD8^+^ T-cells (Fig. 3B). Dilution of CFSE was not detectable in either F5/B6 or F5/LΔP CD8^+^ T-cells until day 2 post-infection. However, wildtype CD8^+^ T-cells proliferated more than CD8^+^ T cells expressing L∆P L-selectin (Fig. 3C), as evident by CFSE dilution and the significantly higher division index for F5/B6 CD8^+^ T-cells relative to F5/L∆P CD8^+^ T-cells (Fig. 3D). The increased dilution of CFSE was reflected by a striking 8-fold increase in the number of F5/B6 CD8^+^ T-cells in comparison with F5/L∆P T-cells in the mediastinal lymph nodes between days 1 and 2 post-infection (Fig. 3E,F). Neither F5/B6 nor F5/LΔP CD8^+^ T-cells proliferated in the absence of vaccNP inoculation. Activated T-cells exit mediastinal lymph nodes on day 3 after vaccNP infection and become detectable in the peripheral blood and at sites of virus replication^3^. Accordingly, we did not analyse CD8^+^ T-cell in the mediastinal lymph nodes beyond day 2 post-infection.

**Figure 4:**
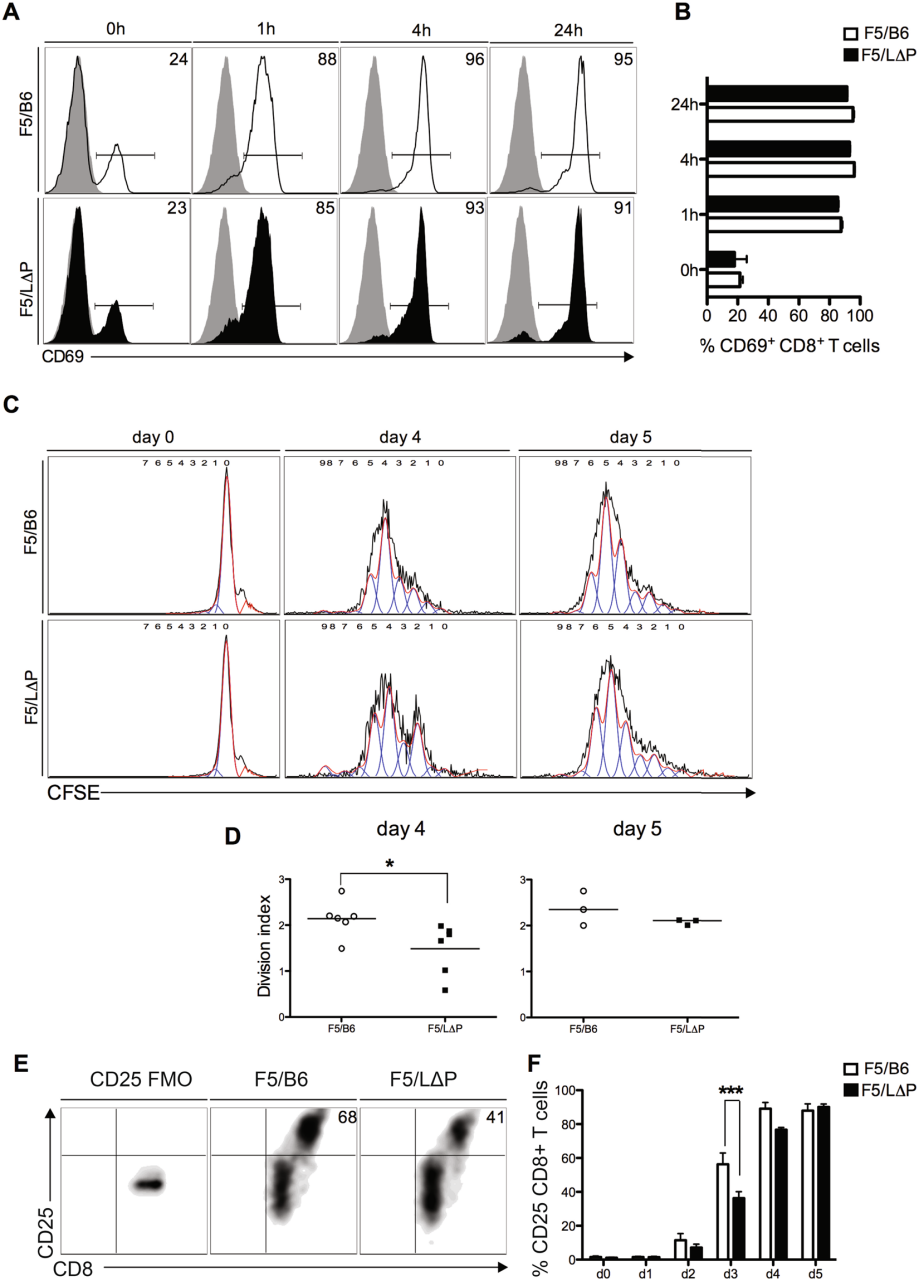
TCR-induced L-selectin shedding promotes CD8^+^ T cell proliferation *in vitro*. (**A**) Representative histograms show CD69 expression on F5/B6 and F5/LΔP CD8^+^ T cells over time after stimulation with cognate peptide-pulsed antigen-presenting cells. Numbers indicate percent CD69^+^ cells. Grey histograms depict staining with an isotype control antibody. (**B**) Bar charts show percent CD69^+^ F5/B6 and F5/L∆P CD8^+^ T cells (mean ± SEM, n = 3). (**C**) Representative histograms show CFSE label in F5/B6 and F5/LΔP CD8^+^ T cells at days 0, 4, and 5 after stimulation *in vitro* with peptide-pulsed antigen-presenting cells. (**D**) Scatter plots of division indices for F5/B6 and F5/LΔP CD8^+^ T cells at days 4 and 5 post-activation *in vitro*. (**E**) Density plots show up-regulation of CD25 by F5/B6 and F5/LΔP CD8^+^ T cells at day 3 post-activation *in vitro*. (**F**) Bar charts show CD25 expression by F5/B6 and F5/LΔP CD8^+^ T cells at days 0, 1, 2, 3, 4, and 5 post-activation *in vitro* (mean ± SEM, n = 5). Statistical significance was assessed using unpaired Student’s 2-tailed t test in panels (D) and (F). *P < 0.05; ***P < 0.001.

To dissect mechanisms linking L-selectin proteolysis to T cell proliferation, F5/B6 and F5/L∆P T cells were stimulated with NP68-pulsed irradiated antigen-presenting cells *in vitro* and analyzed up to 7 days following activation. The kinetics and extent of CD69 upregulation in the first 24 h after TCR engagement were similar in T-cells expressing wildtype or L∆P L-selectin, indicating that ADAM17-mediated L-selectin proteolysis is not required for TCR signal transduction (Fig. 4A,B). To assess the role of L-selectin shedding in T-cell proliferation, F5/B6 and F5/L∆P CD8^+^ T-cells were labelled with CFSE and cultured with peptide-pulsed antigen-presenting cells and exogenous IL-2. After 4 days, wildtype CD8^+^ T-cells proliferated more than CD8^+^ T cells expressing L∆P L-selectin (Fig. 4C), as evident by CFSE dilution and the significantly higher division index for F5/B6 CD8^+^ T-cells relative to F5/L∆P CD8^+^ T-cells (Fig. 4D). Comparison of CFSE dilution profiles showed a transient delay in proliferation that was restricted to a subpopulation of F5/L∆P CD8^+^ T-cells, which resolved by day 5.

F5/B6 and F5/L∆P CD8^+^ T-cell cultures were supplemented equally with exogenous IL-2 on the second day after stimulation and we have reported that F5/B6 and F5/L∆P CD8^+^ T-cells synthesise similar levels of IL-2 *in vitro*^3^. Therefore, we reasoned that differences in proliferation were not related to cytokine levels and instead determined the role of receptors for IL-2. We started by analysing the expression of CD25 (IL-2Rα) which together with CD127 (IL-2Rβ) and CD132 (IL-2Rγ) forms a high affinity receptor for IL-2^31^. In the absence of activation, ~2% of F5/B6 and F5/L∆P CD8^+^ T-cells expressed CD25. The frequency of CD25 expressing F5/B6 CD8^+^ T-cells started to increase by day 2 following peptide-MHC stimulation and increased to maximal levels by day 4, when ~90% of cells expressed CD25. CD25 expressing F5/L∆P CD8^+^ cells also increased between days 2 and 4 following TCR-activation. However, the frequency of CD25 expressing F5/L∆P CD8^+^ was consistently lower than CD25^+^ F5/B6 T cells at days 2, 3 and 4 and the difference was statistically significant at day 3 when 60% of F5/B6 CD8^+^ T-cells expressed CD25, whereas only 38% of F5/L∆P CD8^+^ T-cells expressed CD25 (Fig. 4E,F). Differences in CD25 expression between F5/B6 and F5/LΔP T cells were no longer seen at day 5 when ~90% of both cell types expressed CD25. The delay in proliferation of F5/L∆P CD8^+^ T-cells correlated with reduced expression of CD25.

Metalloproteinases such as matrix metalloproteinase-9 (MMP9) have been shown to regulate CD25 expression on tumour infiltrating T cells in clinical cancers^32^. We tested whether the level of CD25 on activated F5 T cells is regulated by metalloproteinases and included GW or GI for 24 hours from day 2 to day 3 of T cell activation when CD25 expression is upregulated. CD25 expression was not affected by GW or GI (MFIs: Control 637 ± 3; GI: 598 ± 30; GW: 672 ± 56, n = 3) showing no role for ADAM17 or ADAM10 in controlling CD25 expression by activated CD8^+^ T cells. The lack of effect of either GW or GI also eliminates roles for MMP3, MMP9 or MMP13, which are also inhibited by GI and/or GW^27^, in controlling CD25 expression in activated CD8^+^ T cells. The impact of a delay in proliferation of F5/L∆P CD8^+^ T-cells as shown by CFSE dilution was detectable on day 4 and maintained during the subsequent rapid expansion of T cells by the increased numbers of F5/B6 over F5/L∆P CD8^+^ T-cells of 4.9-fold, 5.5-fold and 3.7- fold on days 4, 6 and 7 respectively following stimulation.

Our findings of earlier and increased clonal expansion of isolated T cells able to shed L-selectin are consistent with findings in virus-infected mice. The earlier clonal expansion of CD8^+^ T-cells in mice is striking in comparison with peptide-MHC stimulated T cell proliferation *in vitro*. This is due, in part, to the experimental design in that isolated F5 CD8^+^ T cells are stimulated by cognate peptide and supplemented with cytokines on day 2 after T cell stimulation whereas in lymph nodes, F5 CD8^+^ T cells are supplemented with cytokines derived from polyclonal CD4^+^ T helper cells also activated by virus. Other contributing factors may be the induction of chemokine dependent recruitment of T cells inside lymph nodes which optimizes T cell priming by virus-infected antigen presenting cells^33^ and/or differences in peptide loading onto MHC by infectious virus. Collectively, our findings demonstrate that ADAM17-dependent, L-selectin proteolysis following engagement of the TCR plays a critical role in early clonal expansion of cytotoxic CD8^+^ T-cells, potentially via regulation of CD25 expression. Further studies will be required to determine how L-selectin cleavage affects CD25 expression and proliferation in T cells. The dominant role of L-selectin in controlling leucocyte adhesion has focused attention on its adhesive function. The cytoplasmic tail of L-selectin binds to a number of structural and signalling proteins known to regulate leucocyte adhesion^34,35^. The soluble fragment of L-selectin retains ligand binding activity and acts as an adhesion buffer by limiting leucocyte recruitment from the bloodstream^16^. The impact of L-selectin cleavage of T cell proliferation could be an indirect effect of altered binding to antigen presenting cells which express PSGL-1^36^, a cognate ligand for L-selectin, resulting in altered transmission of co-stimulatory signals to T cells. However, TCR engagement stimulates phosphorylation-dependent binding of protein kinase C isozymes ι, θ, and α to the cytoplasmic tail of L-selectin^8^, some of which are known to regulate T cell activation. It is, therefore, possible that the membrane associated cleaved fragment directly stimulates T cell proliferation or that non-cleaved L-selectin inhibits T cell proliferation due to lack of translocation to a different membrane compartment. Further experiments will be required to pin-point the underlying mechanism or mechanisms.

The presented findings predict that, in the absence of L-selectin shedding, reduced clonal expansion of cytotoxic T cells in lymph nodes will affect the kinetics of virus clearance in peripheral tissues if the number of cytotoxic T cells generated is insufficient to kill virus-infected cells. We have already demonstrated the pathophysiological importance of L-selectin proteolysis in memory CD8^+^ T cells in that T cells unable to shed L-selectin show reduced viral clearance^37^. The delayed clonal expansion of cytotoxic T cells unable to shed L-selectin reported here is a potential explanation for reduced clearance of virus by memory T cells unable to shed L-selectin. Mouse T cells expressing a different construct of L-selectin in which the membrane proximal region of E-selectin is knocked into wildtype L-selectin, have been shown to resist shedding stimulated by anti-CD3 antibodies but the impact on T cell proliferation was not determined^17^. Interestingly, T cells unable to shed L-selectin due to a deficiency in ADAM17 showed no deficit in CD4^+^ or CD8^+^ T-cell activation following bacterial infection^13^. This suggests that ADAM17-dependent proteolysis of L-selectin may not control T cell activation or function during bacterial infection. However, other ADAM17 substrates known to control T cell activation such as IL6Rα^13^ or LAG-3^14^ will not be proteolyzed in ADAM17 null T cells and may mask a role for L-selectin proteolysis in T cell activation. Our approach using T-cell specific cleavable or non-cleavable L-selectin is a conceptual advance that overcomes the severe limitations of targeting ADAM17 in T-cells where the cleavage of up to 70 proteins will be dysregulated, some of which are known to control T-cell activation, thus allowing the analysis of ADAM17 dependent proteolysis of L-selectin in health and disease.

In the T cell lineage, L-selectin identifies naïve and central memory T cell populations both of which have high proliferative potential^38^. L-selectin also marks a stem-like population in the CD8^+^ and NKT lineages which show renewal and persistence^39,40^. Apart from lymph node homing, the contribution of L-selectin to the biology of T cells has not been extensively studied. It has long been known that there is significant cross-talk between L-selectin and TCR signalling but the physiological relevance of TCR-induced downregulation of L-selectin during T-cell activation has remained elusive. Based on our findings, the potential of ADAM17 dependent L-selectin proteolysis in regulating the activation of other types of leucocyte in disease settings should be considered, even in cells recorded as L-selectin negative at the time of analysis due to active ADAM17. In this manuscript we present the novel concept that proteolysis of L-selectin by ADAM17 controls T-cell activation inside lymphoid tissues and present data to show this both in isolated T-cells and in mice. Our results show that cleavage of L-selectin by ADAM17-dependent proteolysis promotes early clonal expansion of cytotoxic T-cells. On this basis, we propose that ADAM17 dependent L-selectin proteolysis should be considered as a regulator of T-cell activation at sites of immune activity.

## Materials and Methods

### Mice

RAG-1-deficient mice were bred at the Frances Crick Institute (London, UK). C57BL/6 (B6) mice were purchased from Harlan or Charles River Laboratories. ADAM17^+/ΔZn^ heterozygous mice were provided by Dr Jacques Peschon and Dr Roy Black (Immunex) and C57BL/6 L-selectin knockout mice by Professor Tom Tedder. L-selectin knockout (CD62L^−/−^) mice expressing a shedding-resistant form of L-selectin as a transgene on either polyclonal T-lymphocytes (LΔP) or CD8^+^ T-cells co-expressing F5 TCR (F5/LΔP) have been described^15^. All other mice were bred in house.

#### ADAM17 sufficient and ADAM17 deficient chimeras

Embryos were collected at e16.5–17 after timed matings of ADAM17^+/ΔZn^ heterozygous mice, in which the zinc-binding domain of ADAM17 was replaced with neomycin^10^ and genotyped for the zinc-binding domain and the neomycin gene using 15 µl of tail tip DNA in separate PCR reactions. Primers (25 pM) for the zinc-binding domain (forward, 5′ CCA CGA GAA TAA TAA GGT ATG TCT 3′; reverse, 5′ AGG AAG AGG AAG GGG ACT A 3′; 360 bp product) or neomycin (forward, 5′ GGA GAG GCT ATT CGG CTA TG 3′; reverse, 5′ CAG GAG CAA GGT GAG ATG A 3′; 281 bp product) were mixed with 200 µl of dNTPs and 1.25 U Taq polymerase in 50 µl of PCR buffer. Reactions were denatured for 5 minutes at 94 °C, DNA amplified over 34 thermocycles (1 minute at 94 °C, 1 minute at 59 °C, and 30 seconds at 72 °C). PCR products were separated on 3% agarose gels containing 0.5 µg/ml ethidium bromide and visualized. ADAM17-deficient (ADAM17^ΔZn/ΔZn^) embryos were identified by the the presence of the neomycin gene, wildtype (ADAM17^+/+^) embryos were identified by the presence of the zinc-binding domain, and heterozygous (ADAM17^+/ΔZn^) embryos were identified by the presence of both the zinc-binding domain and the neomycin gene. In some experiments, eyelid fusion was used to phenotype embryos (open eyelids: ADAM17 deficient; closed eyelids: wildtype and ADAM17 heterozygotes). Fetal liver cells were isolated from ADAM17^ΔZn/ΔZn^ embryos or pooled wildtype ADAM17^+/+^ and heterozygous ADAM17^+/ΔZn^ littermates by passing through 70 µm cell strainers, washed in PBS, resuspended in FCS containing 10% DMSO, and stored at −80 °C. Thawed cells (5–10 × 10^5^) were washed, resuspended in PBS and injected intravenously into sublethally irradiated (5–6 Gy) C57BL/10 RAG-1^−/−^ or C57BL/6 L-selectin^−/−^ mice. Chimeric mice were tested for ADAM17 based on susceptibility or resistance to PMA-induced shedding of L-selectin on peripheral blood T-lymphocytes^15^. Lymphocytes generated from genotyped (n = 6) and phenotyped (n = 12) ADAM17^ΔZn/ΔZn^ embryos failed to shed L-selectin in response to PMA^10^. PMA-induced shedding was equivalent in ADAM17 sufficient lymphocytes (ADAM17^+/+^ and ADAM17^+/ΔZn^)^10^ and data generated using ADAM17^+/+^ and ADAM17^+/ΔZn^ lymphocytes were pooled and described as ADAM17^WT^ lymphocytes. All experiments were conducted according to institutional guidelines and UK Home Office regulations using age/sex-matched mice aged 8–12 weeks. The genotypes of mouse strains are summarized in Supplementary Table 1.

### L-selectin-expressing lymphoid cells

Peripheral lymph nodes (axillary, brachial, and inguinal) from ADAM17 chimeras, LΔP, F5/LΔP, B6, or F5/B6 mice, all aged 12–16 weeks, were collected into Ca^2+^/Mg^2+^-free PBS on ice, passed through 70 µm cell strainers, and washed in PBS. Lymphocytes were collected by centrifugation at 250 g. CD8^+^ T-cells co-expressing the F5 transgenic TCR and either wildtype L-selectin (F5/B6) or shedding-resistant L-selectin (F5/LΔP) were isolated from pooled spleen and peripheral lymph node samples by negative selection using a CD8α^+^ T-cell isolation kit with LS columns (Miltenyi Biotec). The genotypes of mouse T-cells are summarized in Supplementary Table 2.

Human L-selectin negative MOLT-3 acute lymphoblastoid T-cells (ATCC CRL-1552), were sequentially transduced with lentiviral particles isolated from transiently transfected 293 T cells to express an HIV-1 Gag SLYNTVATL-specific TCR (868)^41^ and either wildtype L-selectin or shedding-resistant ∆M-N human L-selectin^26^. Full-length and ∆M-N-L-selectin were amplified using PfuUltra II Fusion HS DNA Polymerase (Agilent), and the PCR product was cleaved with BamHI and XhoI before ligation into BamHI-linearized pSxW using In-fusion (Clontech). Briefly, 2 × 10^7^ 293 T cells were incubated overnight in T175 flasks (Thermo Fisher Scientific). The following day, medium was replaced with 12 ml of DMEM supplemented with 10% FCS (pH 7.9). Transfection mix (3 ml) containing 60 μg of pSxW, 60 μg of pCMVΔ8.91, 30 μg of pMD2G^42^, and 0.15 M CaCl_2_ in serum-free DMEM (pH 7.1) was added dropwise, and the flasks were incubated overnight at 37 °C. Medium was replaced after 24 hours with 20 ml of fresh DMEM 10% FCS. Lentiviral particles were collected at 48 and 72 hours post-transfection. Supernatants were ultracentrifuged using a Sorvall SW28 rotor at 26,000 rpm for 2 h, concentrated 10-fold, and stored at −80 °C. MOLT-3 cells (0.5 × 10^6^) were transduced with concentrated lentiviral particles and 4 μg/ml Polybrene (Sigma-Aldrich). Cells were analyzed for transgene expression by flow cytometry 48 h after transfection. Leukemic T-cell clones were isolated by limiting dilution and grown in complete RPMI 1640 medium 10% FCS (R10).

### L-selectin shedding

ADAM17^ΔZn/ΔZn^, ADAM17^WT^, L∆P and C57BL/6 mice were injected intraperitoneally with 10 µg of SEB (Sigma-Aldrich). After 4 h, pooled peripheral lymph nodes (inguinal, brachial, and axillary) were collected and stained for L-selectin and Vβ8^25^.

C57BL/6 mice were injected intravenously with 2 × 10^6^ CD8^+^ T-cells co-expressing the F5 transgenic TCR and either wildtype (F5/B6) or LΔP L-selectin (F5/LΔP). Next day, mice were injected intraperitoneally with 2 × 10^6^ plaque-forming units (pfu) of recombinant vaccinia virus, expressing the nucleoprotein peptide ASNENMDAM (NP68) from influenza A strain E61-13-H17 (vaccNP). Draining mediastinal lymph nodes were harvested at 24 h and 48 h and analyzed for donor F5 CD8^+^ T-cell expression of L-selectin and CD69^3^.

Blood was collected from the tail veins of ADAM17^ΔZn/ΔZn^, ADAM17^WT^, L∆P, and C57BL/6 mice directly into heparinized capillary tubes (Sigma-Aldrich). Whole blood (40 µl) was incubated with 300 nM PMA dissolved in DMSO or an equivalent volume of DMSO vehicle control for 45 minutes at 37 °C. Red blood cells were then lysed, and L-selectin expression quantified by flow cytometry. Plasma was collected by centrifugation. Soluble mouse L-selectin was quantified by ELISA^15^ or by using a Mouse sL-Selectin/CD62L Quantikine ELISA Kit (R & D Systems).

Basal and PMA-induced L-selectin shedding was measured in lymph node cells, resuspended at 5 × 10^7^/ml in RPMI 1640 supplemented with 1% FCS (R1). Aliquots of 50 µl (2.5 × 10^6^ cells) were incubated for 1 h at 4 °C or 37 °C in the presence of 300 nM PMA and/or the broad spectrum ADAM/MMP inhibitor Ro 31–9790 (both dissolved in DMSO) or an equivalent volume of DMSO as vehicle control^15^. T-cells were analysed for cell surface L-selectin by flow cytometry, and supernatants analysed for soluble L-selectin by ELISA.

TCR-induced shedding of L-selectin in F5/LΔP and F5/B6 mice was measured using CD8^+^ T-cells resuspended in DMEM 10% FCS, penicillin-streptomycin, L-glutamine, non-essential amino-acids, and β-mercaptoethanol. Splenocytes from B6 mice were pulsed with 5 µg/ml NP68 peptide (Peptide Synthetics) for 1 h at 37 °C and irradiated at 30 Gy. F5/B6 and F5/LΔP CD8^+^ T cells (2 × 10^6^ cells/well) were incubated with splenocytes (6 × 10^6^ cells/well) at 37 °C in 24-well plates (Nunclon). Fresh complete medium supplemented with 360 IU/ml hrIL-2 was added on day 2. Cells were harvested as indicated, stained for CD8 and L-selectin by flow cytometry.

TCR-induced L-selectin shedding in MOLT-3 cells was examined in response to HLA-A2^+^ C1R B cells pulsed with SLYNTVATL (SLY) peptide (Eurofins). MOLT-3 cells and C1R cells were rested overnight at 0.9 × 10^6^ cells/ml in RPMI 1640 supplemented with 2% FCS (R2). The following day, C1R cells were resuspended at 0.5 × 10^6^ cells/ml in the absence or presence of SLY peptide in R2 and distributed at 0.5 × 10^5^ cells/well in 96-well U-bottomed plates. Cells were incubated for 1 h at 37 °C and washed in R2. MOLT-3 cells were resuspended at 1.5 × 10^6^ cells/ml and added to C1R cells at a ratio of 3:1. The plates were incubated for 1 h at 37 °C. To study the effect of shedding inhibitors, MOLT-3 cells were pre-incubated with GW, GI, or D1(A12)^29^ for 30 minutes, added to SLY peptide-pulsed C1R cells in R2 containing inhibitors. Cells were washed, stained, and analysed by flow cytometry.

#### TCR-induced T cell proliferation

To assess the impact of L-selectin proteolysis on CD8^+^ T cell proliferation, 10^7^ naïve F5/B6 or F5/LΔP CD8^+^ T-cells were resuspended in 1 ml of PBS and labelled with 5 μM CFSE for 10 minutes at room temperature in the dark. The reaction was quenched by adding 10 ml of ice-cold FCS. Cells were then washed extensively in ice-cold PBS, resuspended in PBS, and injected intravenously at 2 × 10^6^ cells/mouse. After 24 h, mice were inoculated intraperitoneally with 2 × 10^6^ pfu vaccNP. Lymphoid tissues were harvested and analysed for donor cell proliferation by quantification of CFSE dilution on days 1/2 post-infection.

Virus-specific CD8^+^ T-cell proliferation was also assessed via incorporation of 5-ethynyl-2-deoxyuridine (EdU) (Life Technologies). Mice were injected intraperitoneally with 1 mg of EdU. Organs were harvested the following day and processed as single-cell suspensions. Cells were stained for extracellular markers, fixed with 4% formaldehyde, permeabilized in saponin buffer, and analysed for incorporation of EdU using a Click-it Plus EdU Alexa Fluor 647 Flow Cytometry Assay Kit (Life Technologies).

CD8^+^ T-cell proliferation in response to peptide antigen stimulation was assessed via CFSE dilution. Naïve F5/B6 or F5/LΔP CD8^+^ T-cells were labelled with 2 μM CFSE, washed extensively in ice-cold PBS, resuspended in complete DMEM, and plated at 2 × 10^6^ cells/well in 24-well plates (Nunclon). Splenocytes from F5/B6 mice were pulsed with 5 µg/ml NP68 peptide (Peptide Synthetics) for 1 h at 37 °C, irradiated at 30 Gy and added at 6 × 10^6^ cells/well. The plates were then incubated at 37 °C. Fresh complete medium supplemented with 360 IU/ml hrIL-2 was added on day 2. Cells were harvested as indicated and CFSE dilution was analyzed by flow cytometry.

In some experiments, F5 homozygous CD8^+^ T cells^43^ were cultured with NP68 peptide pulsed splenocytes or with 200 µg/ml of anti-CD3 (145.2C11; BD Biosciences) and 100 µg/ml anti-CD28 (37.51 BD Biosciences), culture media supplemented with IL2 plus 3 µM GI, 3 µM GW or equivalent volume of vehicle control (DMSO) on day 2 and CD8^+^ T cells analysed for CD25 expression on day 3.

#### Flow cytometry

For surface staining, cells were washed in PBS 2% FCS, labelled with Live/Dead Fixable Aqua (Life Technologies), washed in PBS 2% FCS, and blocked with PBS 2% FCS containing 5% rat serum. Cells were stained with relevant antibodies for 30 minutes at 4–8 °C, washed in PBS 2% FCS, fixed with 4% paraformaldehyde, and resuspended after a wash in 200 μl PBS supplemented with 2% FCS. Samples were acquired using a FACS Canto II flow cytometer (BD Biosciences) and CytoCount beads were used to quantify cell numbers, according to the manufacturer’s instructions (DakoUK Ltd, Ely, UK). Data were analyzed using FlowJo version 9.7.6 (Tree Star).

The following antibodies were used to stain mouse T-cells: anti-Vβ11-FITC (KT-11), anti-CD8-PerCPCy5.5 (53–6.7), anti-CD25-Pacific blue (JES6-5H4), anti-CD44-APCCy7 (IM7), anti-CD62L-biotin, anti-CD62L-FITC, or anti-CD62L-PECy7 (MEL-14), anti-CD69-APC (H1.2F3), anti-CD90.1-PECy7 (OX-7), anti-CD90.2/Thy1.2-Pacific Blue (53-2.1) or anti-CD90.2-Alexa Fluor 488 (30-H12) (BioLegend), anti-Vβ8-FITC (F23.1) (BD Biosciences), and anti-TCR-PE (H57-597) (Southern Biotechnology). Fluorescence minus one (FMO) controls were used to detect spreading error. The following antibodies were used to stain human T-cells: anti-CD62L-PE (Dreg 56) with isotype control P3.6.2.8.1-PE (IgG1, κ) (eBioscience); and anti-Vβ5a-FITC (1C1) with isotype control MOPC-31C-FITC (IgG1, κ) (BD Biosciences). C1R cells in mixed cultures were identified using anti-CD19-APC (H1B18) (BD Biosciences) with isotype control 11711-APC (IgG1, κ) (R & D Systems). L-selectin expression was calculated relative to vehicle-treated cells after subtracting the isotype or FMO median fluorescence intensity as follows:

$$ \% L \mbox{-} {\rm{selectin}}=\frac{({\rm{MFI}}\,{\rm{sample}}-{\rm{MFI}}\,{\rm{isotype}}\,{\rm{or}}\,\mathrm{FMO})}{(\mathrm{MFI}\,{\rm{untreated}}\,{\rm{cells}}-\,{\rm{MFI}}\,{\rm{isotype}}\,{\rm{or}}\,\mathrm{FMO})}\times 100$$

### Statistical analysis

The number of animals and replicates are indicated in each figure legend. All data are presented as mean ± SEM, unless otherwise stated. Statistical analyses were performed using Prism software Mac version 7 (GraphPad) as detailed in figure legends. Figures were prepared using FlowJo software (Treestar Inc), Prism 7 (GraphPad Software Inc.), MS Powerpoint and Adobe Illustrator. Investigators were blinded to the group allocation during the experiment and drug treatment whenever possible.

### Study Approval

Animal experimental protocols adhered to local and national guidelines. Animal work was conducted inside the designated establishment at Cardiff University which fully complies to the Home Office Code of Practice for the Housing and Care of Animals Bred, Supplied or Used for Scientific Purposes pursuant to the Animal (Scientific Procedures) Act, 1986. All studies were approved by the Animal and Welfare Ethical Review Body at Cardiff University and conducted using project licence 30/3188 under the Animal (Scientific Procedures) Act, 1986.

## Supplementary information

Supplementary Information
