# Supplementary material for: ADAM17-dependent proteolysis of L-selectin promotes early clonal expansion of cytotoxic T cells
Source: Sci Rep. 2019 Apr 2;9:5487. doi: 10.1038/s41598-019-41811-z (PMC6445073; doi:10.1038/s41598-019-41811-z)
Supplement: Supplementary file 1 — Supplementary Information [file 41598_2019_41811_MOESM1_ESM.docx]

**ADAM17-dependent proteolysis of L-selectin promotes early clonal expansion of cytotoxic T cells**

Rebar N Mohammed^1,2^*, Sophie C Wehenkel^1^*, Elena V Galkina^3,4^, Emma-Kate Yates^3^, Graham Preece^3,^ , Andrew Newman^1^, H Angharad Watson^1^, Julia Ohme^1^, John S Bridgeman^1^, Ruban R P Durairaj^1^, Owen R Moon^1^, Kristin Ladell^1^, Kelly L Miners^1^, Garry Dolton^1^, Linda Troeberg^5^, Masahide Kashiwagi^6^, Gillian Murphy^7^, Hideaki Nagase^8^, David A Price^1,9^, R James Matthews^1^, Vera Knäuper^10^ and Ann Ager^1,9^

* These authors contributed equally to the manuscript.

*This paper is dedicated to Graham Preece who died on 11^th^ November 2018.*

^1^Divsion of Infection and Immunity, School of Medicine, Cardiff University, Cardiff CF14 4XN, UK.

^2^College of Veterinary Medicine, University of Sulaimani, Sulaimani, Kurdistan/Iraq.

^3^Francis Crick Institute, London, NW1 1AT, UK.

^4^Department of Microbiology and Molecular Cell Biology, Eastern Virginia Medical School, Norfolk, VA 23507, USA.

^5^Norwich Medical School, University of East Anglia, Norwich, NR4 7UQ, UK.

^6^Takeda Pharmaceutical Research Institute, Tsukuba, Japan.

^7^University of Cambridge Department of Oncology, Cancer Research UK Cambridge Institute, Li Ka Shing Centre, Cambridge, CB2 0RE, UK.

^8^Kennedy Institute of Rheumatology, University of Oxford, Oxford OX3 7FY, UK.

^9^Systems Immunity Research Institute, Cardiff University, Heath Park, Cardiff CF14 4XN, UK.

^10^School of Dentistry, Cardiff University, Heath Park, Cardiff CF14 4XN, UK.

**Corresponding author**: agera@cardiff.ac.uk.

**
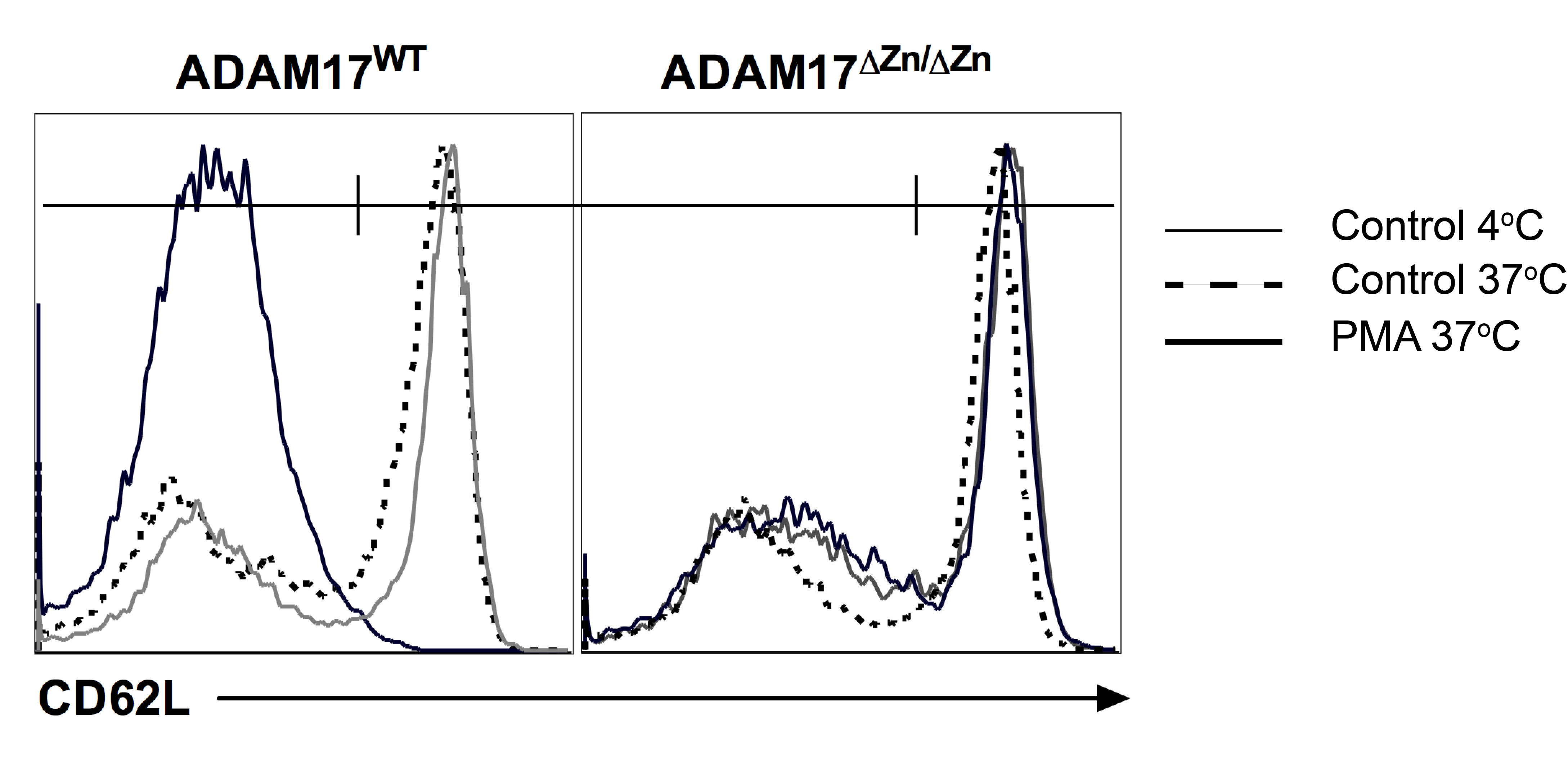
**

**Supplementary Figure 1, related to Figure 1. Effect of ADAM17 on L-selectin expression by T-cells under homeostatic and activating conditions.**

ADAM17-sufficient (ADAM17^WT^) and ADAM17-deficient (ADAM17^ΔZn/ΔZn^) T-cells harvested from peripheral lymph nodes of RAG-1-deficient haematopoietic stem cell chimeric mice were incubated at 4 °C or 37 °C in the absence or presence of 300nM PMA. Cell surface expression of L-selectin (CD62L) was analyzed by flow cytometry. Representative histograms show overlays of treatments and gating of L-selectin (CD62L) positive T-cells.

| **Mouse ID** | **ADAM17** | **Background** | **F5 TCR** | **Wildtype L-selectin** | **LΔP transgene** | **Thy** |
| --- | --- | --- | --- | --- | --- | --- |
| ADAM17^WT^ | +/+ or +/- | B6 RAG-1^-/-^ chimera | none | +/+ | none | Thy1.2 |
| ADAM17^ΔZn/ΔZn^ | -/- | B6 RAG-1^-/-^ chimera | none | +/+ | none | Thy1.2 |
| ADAM17^WT^ | +/+ or +/- | B6 L-selectin^-/-^chimera | none | +/+ | none | Thy1.2 |
| ADAM17^ΔZn/ΔZn^ | -/- | B6 L-selectin^-/-^chimera | none | +/+ | none | Thy1.2 |
| DBA | +/+ | DBA | none | +/+ | none | Thy1.2 |
| ADAM17^ΔZn/ΔZn^  DBA | -/- | DBA | none | +/+ | none | Thy1.2 |
| F5/B6 | +/+ | B6 | hemizygous | +/+ | none | Thy1.2 |
| F5/LΔP | +/+ | B6 | hemizygous | -/- | hemi-  zygous | Thy1.2 |
| LΔP | +/+ | B6 | none | -/- | hemi-  zygous | Thy1.2 |
| C57BL/6 (B6) | +/+ | B6 | none | +/+ | none | Thy1.2 |
| Thy1.1 | +/+ | B6 | none | +/+ | none | Thy1.1 |

**Supplementary Table 1. Genotype of mouse strains used.**

| **Cell line ID** | **Species** | **Cell type** | **TCR** | **Wildtype L-selectin** | **L-selectin transgene** |
| --- | --- | --- | --- | --- | --- |
| L-selectin | Human | Molt3 | 868 | -/- | Wildtype human L-selectin |
| ΔM-N | Human | Molt3 | 868 | -/- | Shedding-resistant human L-selectin |
| F5/B6 | Mouse | CD8^+^ naïve or CTL | F5 | +/+ | none |
| F5/LΔP | Mouse | CD8^+^ naïve or CTL | F5 | -/- | Shedding-resistant mouse L-selectin |

**Supplementary Table 2. Genotype of T cells used.**
